# Inter-subject receive-template substitution for quantitative brain water content mapping at 3 T: a leave-one-out evaluation

**DOI:** 10.3389/fnins.2026.1986813

**Published:** 2026-10-19

**Authors:** Zaheer Abbas, Faizan Ullah, Sergo Gegechkori, Wieland A. Worthoff, N. Jon Shah

**Affiliations:** 1 Institute of Neuroscience and Medicine 4, Medical Imaging Physics, Forschungszentrum Jülich, Jülich, Germany; 2 Department of Neurology, RWTH Aachen University, Aachen, Germany; 3 JARA–BRAIN – Translational Medicine, Aachen, Germany

**Keywords:** brain water content, inter-subject template, leave-one-out evaluation, proton density mapping, quantitative MRI, receive sensitivity correction

## Abstract

**Introduction:**

To evaluate a configuration-specific inter-subject receive template as an alternative to participant-specific receive referencing within the evaluated 3 T scanner configuration.

**Methods:**

Datasets from twenty-five healthy subjects were analyzed using a strict leave-one-participant-out method. Each degree-3 template used the other 24 participants. Arm E used a participant-specific effective relative receive divisor; Arm T used the held-out template. Both arms underwent independent, identically defined residual calibration, including a local intercept (C_0_), its spatially regularized field (μ), and the mean-normalized form used downstream (μ). This was followed by ventricular-CSF calibration. Primary outcomes were participant-level median quantitative water content (qWC) in white and gray matter; whole brain was secondary. Paired bias, mean absolute error (MAE), Bland–Altman limits, Pearson r, and Lin concordance correlation coefficient (CCC) were calculated.

**Results:**

Mean relative receive-template mismatch was 2.273% (standard deviation (SD), 0.568%; range, 1.431%–3.615%). Mean Arm T minus Arm E differences were −0.04 percentage units (p.u.; dimensionless water fraction relative to pure water = 100 p.u.) in white matter and −0.17 p.u. in gray matter; MAE was 0.17 and 0.22 p.u., and CCC was 0.991 and 0.989, respectively. Whole-brain bias was −0.12 p.u. (MAE, 0.19 p.u.; CCC, 0.993). Bland–Altman widths were below 1 p.u. Exploratory associations between global mismatch and qWC disagreement were weak; all 95% participant-bootstrap confidence intervals spanned zero.

**Discussion:**

Under symmetric arm-specific calibration, the configuration-specific template produced qWC estimates in close agreement with the participant-specific explicit reference strategy. This internal matched-configuration result supports technical feasibility but does not establish formal equivalence, interchangeability, or external generalization.

## 1 Introduction

Brain water content is tightly regulated, and changes of only a few percentage points can accompany cerebral edema, inflammation, neurodegeneration, and other pathological conditions (Neeb et al., 2006, 2008). For measurement context, a single-volunteer 3 T study reported across-scan tissue-mean water-content standard deviations of 0.33 p.u. in white matter and 0.38 p.u. in gray matter (Oros-Peusquens et al., 2019). Here, p.u. denotes absolute percentage units of water content rather than relative percent change. Quantitative water content (qWC) mapping with MRI estimates tissue water content from proton density (PD) after correction for relaxation and radiofrequency-field effects and calibration to cerebrospinal fluid (CSF) (Volz et al., 2012a; Abbas et al., 2014).

Retrospective or archived datasets may not contain the matched phased-array/body-coil (PA/BC) reference pair, and a missing participant-specific reference cannot be acquired after the examination; otherwise-compatible datasets may therefore be unable to use explicit receive referencing. At 3 T, phased-array receive sensitivity is a smooth, participant- and position-dependent modulation. In the explicit strategy evaluated here, matched PA and BC acquisitions are processed to derive a participant-specific smooth receive divisor. The pair adds approximately 1.1 min of acquisition time (nominal, 64.0 s; DICOM mean, 64.18 s; range, 63.44–65.08 s) and requires participant-specific receive-field derivation; these additional steps may be undesirable in time-constrained, large-cohort, or clinical workflows. Participant-image-derived corrections based on longitudinal relaxation time (*T*1) or pseudo-proton density instead rely on tissue or intensity assumptions that can make biological water or proton-density variation difficult to distinguish from receive-related bias when that variation is itself the quantity of interest (Volz et al., 2012b; Abbas et al., 2014, 2015). Once established from matched-reference data for a specific scanner/receive-coil configuration, an inter-subject template avoids the participant-specific reference acquisition and derivation in subsequent compatible datasets. This benefit comes with an initial configuration-specific establishment requirement, loss of participant- and position-specific adaptation, and no assumed transfer across scanner or receive-coil configurations.

Related strategies include population templates for transmit-field inhomogeneity, generic image-adaptive smooth-bias estimation, single-scan qWC protocols, and multichannel algebraic separation. These address different inverse problems and are not equivalent to the effective receive-template substitution evaluated here (Vovk et al., 2007; Castro et al., 2010; Tustison et al., 2010; Mezer et al., 2016; Cordes et al., 2017; Oros-Peusquens et al., 2019).

Template-based correction has previously been explored most extensively for transmit-field inhomogeneity. Applying the concept to receive modulation requires a configuration-specific target and a design that prevents the evaluated participant from contributing to the template. We therefore tested whether a fixed inter-subject effective-relative receive template could substitute for a participant-specific receive-reference acquisition within independently CSF-calibrated qWC using a strict leave-one-participant-out comparison with the explicit-reference strategy. To our knowledge, an inter-subject receive template has not previously been evaluated as a substitute for a participant-specific receive reference in quantitative water content mapping under leave-one-participant-out isolation (Castro et al., 2010).

This study evaluates this substitution within an established dual-flip qWC estimation framework. For the present comparison, the participant-specific explicit-reference strategy is denoted as Arm E, and the leave-one-out template strategy is Arm T; Arm E is a body-coil-referenced relative phased-array receive strategy, not the ground truth. Related quantitative MRI approaches—such as the mrQ framework, quantitative rapid gradient-echo (QRAGE), magnetic resonance fingerprinting, and echo-planar time-resolved imaging—provide context for the broader family of quantitative protocols but were not used as comparator arms because they address different acquisition and quantitative-estimation problems (Ma et al., 2013; Mezer et al., 2013; Abbas et al., 2014; Oros-Peusquens et al., 2019; Wang et al., 2019; Zimmermann et al., 2025).

The primary objective was to estimate the Arm-T-minus-Arm-E difference in participant-level median qWC for white matter and gray matter; whole brain was secondary. A separate relative receive-field analysis quantified how closely the template reproduced each held-out effective relative receive field. The evaluation was specified as estimation and agreement analysis; no equivalence or noninferiority margin was defined or tested.

## 2 Methods

### 2.1 Study design, participants, and ethics

This was a single-site methodological study on healthy adult volunteers at 3 T. The analysis included datasets from 25 male participants (mean age 26.5 ± 5.3 years; range 21–44 years). For this study, the leave-one-out receive templates and template-substitution comparison were constructed and evaluated separately. Each participant served once as the held-out case, and each template used the remaining 24 participants, with no self-inclusion or outcome-based removal (Figure 1). The study protocol was reviewed and approved by the Ethics Committee of the Medical Faculty, RWTH Aachen University, Aachen, Germany. All procedures were performed in accordance with applicable local regulations and institutional ethical requirements. Written informed consent was obtained from all participants before their inclusion in the study.

**Figure 1:**
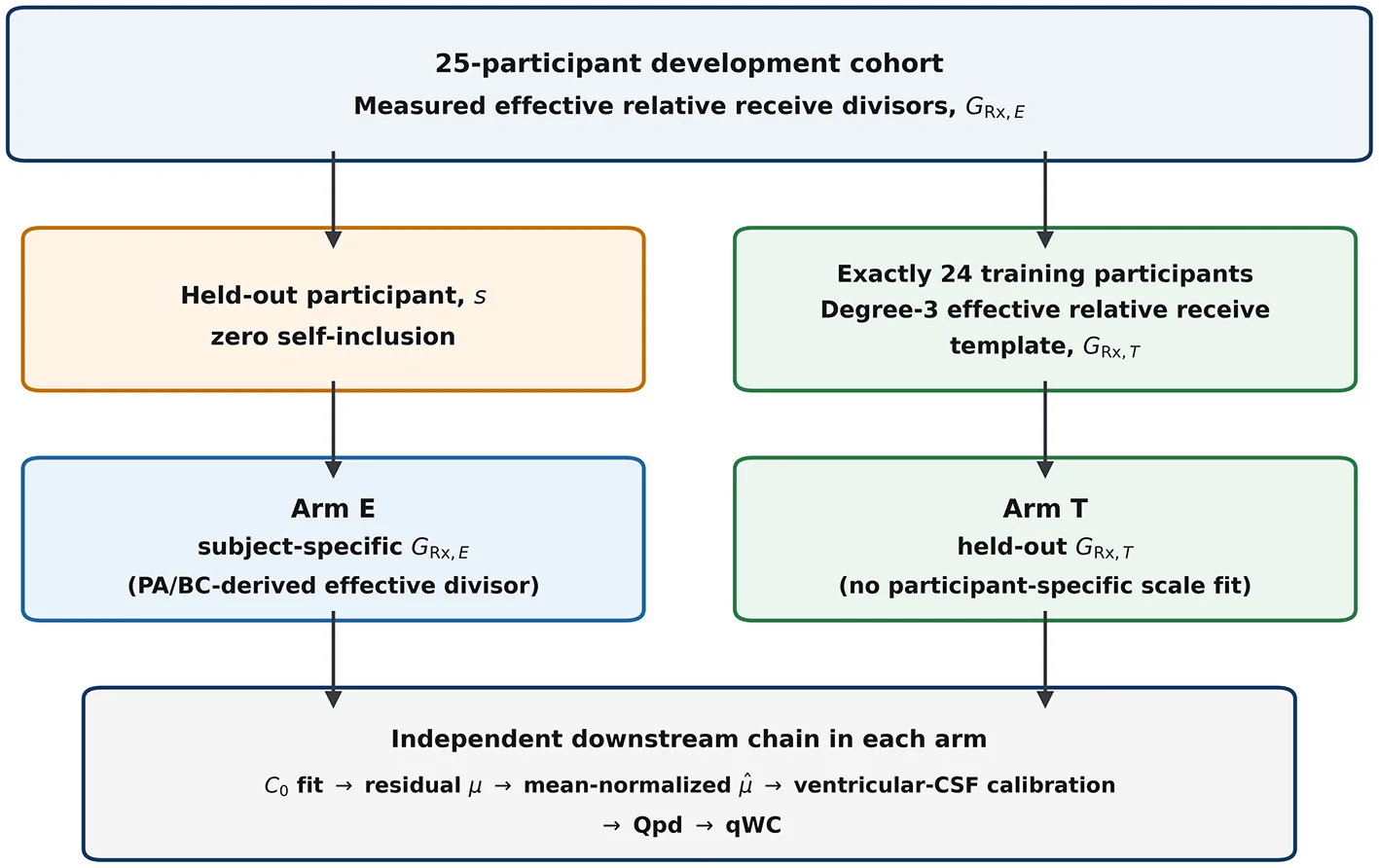
Leave-one-out effective relative receive-template evaluation. For each held-out participant, template coefficients and *G*_Rx,T_ receive-field values came exclusively from the other 24 *G*_Rx,E_ fields. The held-out participant's pre-existing *T*1/SPM-derived brain bounding box defined the spatial evaluation frame; no held-out receive field or qWC outcome entered template fitting, normalization or scale selection. Arm E used participant-specific *G*_Rx,E_; Arm T used held-out *G*_Rx,T_. Each arm underwent independent *C*0, $\mu /\hat{\mu }$ and ventricular-CSF calibration before paired qWC extraction on symmetric common-validity support.

### 2.2 MRI acquisition and hardware

Imaging was performed on a 3 T Siemens Healthineers MAGNETOM Prisma running syngo MR XA30 with a body transmit coil. Phased-array (PA) receive-reference, multi-echo spoiled-gradient-echo low-flip-angle proton-density-weighted (m0w), and geometry-matched higher-flip-angle *T*_1_-weighted (t1w) acquisitions used the HeadNeck_64 receive configuration; the body-coil (BC) receive-reference acquisition used the body receive configuration. The matched PA/BC pair used 2D spoiled gradient echo (matrix, 64 × 64; in-plane resolution, 4 × 4 mm; slice thickness, 2 mm; spacing, 4 mm; repetition time (TR), 500 ms; TE, 5.8 ms; flip angle, 30°; and bandwidth, 260 Hz/pixel; one average). The pair required a nominal 64 s, consistent with Digital Imaging and Communications in Medicine (DICOM) acquisition-to-series timing across the 25 datasets (mean, 64.18 s; range, 63.44–65.08 s). Omitting it therefore removes approximately 1.1 min of acquisition time, excluding setup and transition overhead. The series used to estimate the quantitative maps were m0w, t1w, and a vendor-supplied saturation-prepared turbo fast low-angle shot (satTFL; Siemens sequence identifier tfl2d) ${\text{b}}_{1}^{+}$ map (Chung et al., 2010) (Table 1). Throughout, ${\text{B}}_{1}^{+}$ denotes the physical transmit field and ${\text{b}}_{1}^{+}$ the dimensionless relative transmit factor obtained by normalizing the vendor map to its nominal value; only ${\text{b}}_{1}^{+}$ enters the signal model (Chung et al., 2010).

**Table 1:** Acquisition parameters (3 T Siemens MAGNETOM Prisma; constant across the evaluated cohort).

Parameter	m0w (PD-weighted)	t1w (*T*_1_-weighted)	${\text{b}}_{1}^{+}$ map
Sequence	2D spoiled gradient echo (GRE)	2D spoiled GRE	satTFL (tfl2d)
Nominal flip angle	40°	90°	80°
TR (ms)	1,800	535	18,300
TE (ms)	5.85 (first of 9 echoes)	5.85	2.37
Field of view (mm)	256 × 216	256 × 216	256 × 208
Matrix	256 × 216	256 × 216	128 × 104
Resolution (mm)	1.0 × 1.0 × 1.5	1.0 × 1.0 × 1.5	2.0 × 2.0 × 2.0
Bandwidth (Hz/px)	160	160	698

### 2.3 Inherited T1 and receive-weighted proton-density estimation

*T*1 and receive-weighted proton density were estimated with the established unequal-TR, ${\text{b}}_{1}^{+}$- and slice-profile-corrected framework. This study evaluated receive-correction substitution and did not independently revalidate the upstream *T*1 estimator.

For acquisition *j* in {m0w, t1w}, the local flip angle follows the measured dimensionless relative transmit factor ${\text{b}}_{1}^{+}$, with α expressed in radians (Gras et al., 2013). The relevant signal-model definitions and inversion expressions are provided in Equations 1–7.


$$\begin{array}{l} \alpha_{j}\left(r\right)=b_{1}^{+}\left(r\right)\alpha_{j,nom},j\in \left\{m0w,t1w\right\} \end{array}$$ (1)

$$\begin{array}{l} \beta_{j}=\frac{TR_{j}}{T_{1}} \end{array}$$ (2)

denote the dimensionless ratio of repetition time to *T*_1_, and define the steady-state longitudinal term as


$$\begin{array}{l} s^{-}\left(\alpha_{j},\beta_{j}\right)=\frac{1-e^{-\beta_{j}}}{1-e^{-\beta_{j}}\cos\left(\alpha_{j}\right)} \end{array}$$ (3)

The slice-selective spoiled-GRE magnitude signal is (Gras et al., 2013)


$$\begin{array}{l} |S_{j}|=CM_{0}\sin\left(\alpha_{j}\right)s^{-}\left(\alpha_{j},\beta_{j}\right)\kappa \left(\alpha_{j},\beta_{j}\right)\exp\left(-\frac{TE_{j}}{T_{2}^{*}}\right) \end{array}$$ (4)

Here, *C* contains an acquisition-independent scaling and receive weighting, *M*0 is proton-density-weighted equilibrium magnetization, and κ*(*α,β*)* is the multiplicative slice-profile correction determined by the radiofrequency excitation profile. The measured and theoretical unequal-TR ratios are


$$\begin{array}{l} R_{\text{meas}}=\frac{\left|S_{\text{m}0\text{w}}\right|}{\left|S_{\text{t}1\text{w}}\right|} \end{array}$$ (5)

$$\begin{array}{l} \Gamma \left(T_{1}\right)=\frac{\sin\left(\alpha_{m0w}\right)\left(1-e^{-\beta_{m0w}}\right)\left[1-\cos\left(\alpha_{t1w}\right)e^{-\beta_{t1w}}\right]}{\sin\left(\alpha_{t1w}\right)\left(1-e^{-\beta_{t1w}}\right)\left[1-\cos\left(\alpha_{m0w}\right)e^{-\beta_{m0w}}\right]} \end{array}$$ (6)

and the slice-profile-corrected inversion equation is


$$\begin{array}{l} \Gamma_{2D}\left(T_{1}\right)=\frac{\kappa \left(\alpha_{\text{m}0\text{w}},\beta_{\text{m}0\text{w}}\right)}{\kappa \left(\alpha_{\text{t}1\text{w}},\beta_{\text{t}1\text{w}}\right)}\Gamma \left(T_{1}\right),\Gamma_{2D}\left(T_{1}\right)=R_{\text{meas}} \end{array}$$ (7)

As the first-echo TE was 5.85 ms for both m0w and t1w, the $T_{2}^{*}$ factor cancels in the m0w/t1w ratio.

Receive-weighted proton density was estimated from the low-flip-angle m0w acquisition by dividing excitation, steady-state, and slice-profile terms, with the applicable $T_{2}^{*}$ correction. Phase data used for static-field (B0) processing were unwrapped with ROMEO, and the two-point transmit/receive framework followed the established implementation (Baudrexel et al., 2016; Dymerska et al., 2021).


$$\begin{array}{l} PD_{\text{rx}}\left(r\right)\propto \frac{S_{m0w}\left(r\right)\exp\left(\frac{TE_{\text{m}0\text{w}}}{T_{2}^{*}}\right)}{\sin\left(\alpha_{m0w}\right)s^{-}\left(\alpha_{m0w},\beta_{m0w}\right)\kappa \left(\alpha_{m0w},\beta_{m0w}\right)} \end{array}$$ (8)

### 2.4 Explicit receive-reference construction

For Arm E, matched phased-array (PA) and body-coil (BC) magnitude acquisitions formed the stored effective relative receive divisor *G*_Rx,E_. In the established processing framework, *G*_Rx,E_ is derived from the regularized, median-normalized PA/BC magnitude ratio together with ${\text{b}}_{1}^{+}$ and is interpolated to the quantitative-image lattice. Here, “effective relative receive divisor” means the smooth field used to correct spatial intensity variation caused by the receive coil. It is a practical body-coil-referenced relative strategy, not an absolute physical receive-sensitivity measurement or ground truth (Abbas et al., 2014).

### 2.5 Inter-subject receive-template construction

The arithmetic-mean leave-one-out receive template used the other 24 participants for each holdout. Each training *G*_Rx,E_ field used a brain support defined by summed Statistical Parametric Mapping (SPM25, release 25.01.02) (Ashburner and Friston, 2005) tissue-class probability (gray matter + white matter + CSF) > 0.5, within-support median normalization, and its own brain bounding box followed by first-order interpolation to a 48 × 48 × 32 normalized grid. Finite support was propagated separately. The linear-domain voxel-wise arithmetic mean was formed where defined; polynomial fitting required at least 80% finite training coverage (20/24 fields). A total-degree-3 polynomial in the normalized coordinates [−1, 1] (20 coefficients) was fitted by a single ordinary least-squares solve and evaluated as *G*_*Rx,T*_ in the held-out participant's normalized frame. No ridge regularization, iteratively reweighted least-squares fitting, log-domain fitting, final template normalization, or held-out outcome scaling was used. During method development, the arithmetic-mean construction was selected from candidate template families using leave-one-out receive-field fidelity in the same 25-participant cohort before any Arm-T qWC outcome was generated; no qWC outcome informed construction-family selection. The held-out participant contributed no receive-field data to template construction; only the participant's native brain bounding box was used to define the deployment and evaluation frame. Template coordinates were brain-relative: each native array-index brain bounding box was mapped to [−1, 1]^3^ before resampling and fitting, and the fitted polynomial was evaluated within the held-out participant's own array-index bounding box. This normalization provided a common frame for pooling approximately corresponding brain-relative locations across participants without scanner-space registration, but it did not retain absolute scanner translation, physical head or brain size, voxel spacing, obliquity, DICOM position/orientation, or exact coil-relative head position. Copying the held-out native affine and header to the deployed output did not make scanner coordinates part of the template fit; absolute position- and scale-dependent receive variation could therefore remain as residual mismatch (Supplementary Figure S1).

For each participant, the measured *G*_Rx,E_ and template *G*_Rx,T_ fields were compared on the two-voxel-eroded brain support intersected with finite *G*_Rx,E_, finite *G*_Rx,T_, and positive *G*_Rx,T_. Both fields were independently median-normalized on that common support; mismatch was the median voxel-wise absolute percentage difference using the normalized template in the denominator. Across participants, mismatch was summarized by the cohort mean, sample SD, median, and range. In the equations below, *E*_*s*_ and *T*_*s*_ denote the measured *G*_Rx,E_ and template *G*_Rx,T_ fields of participant *s* on the common support Ω_*s*_, and a tilde marks each field after median normalization on that support.


$$\begin{array}{l} \Omega_{s}={\text{erode}}^{2}\left(M_{\text{brain},\text{s}}\right)\cap \text{finite}\left(E_{s}\right)\cap \text{finite}\left(T_{s}\right)\cap \left\{T_{s}>0\right\}\\ {\overset{\sim }{E}}_{s}\left(v\right)=\frac{E_{s}\left(v\right)}{{\text{median}}_{u\in \Omega_{s}}E_{s}\left(u\right)},{\overset{\sim }{T}}_{s}\left(v\right)=\frac{T_{s}\left(v\right)}{{\text{median}}_{u\in \Omega_{s}}T_{s}\left(u\right)}\\ e_{s}={\text{median}}_{v\in \Omega_{s}}\left(100\frac{\left|{\overset{\sim }{E}}_{s}\left(v\right)-{\overset{\sim }{T}}_{s}\left(v\right)\right|}{\left|{\overset{\sim }{T}}_{s}\left(v\right)\right|}\right)\% \end{array}$$

### 2.6 Arm-specific receive correction and CSF calibration

Arm E used *G*_Rx,E_; Arm T substituted the held-out *G*_Rx,T_, where both *G*_Rx,E_ and *G*_Rx,T_ represent relative receive fields that are independently median-normalized prior to template substitution. Let *P* denote the receive-weighted precursor of Equation 8 (*PD*_rx_) before receive division, and let PDC denote the arm-specific receive-corrected proton-density proxy. As the retained Arm-E PDC already contained division by *G*_Rx,E_, the implementation performed the algebraic substitution.


$$\begin{array}{l} \begin{array}{c} {\text{PDC}}_{E,s}\left(v\right)=\frac{P_{s}\left(v\right)}{G_{\text{Rx},\text{E},\text{s}}\left(v\right)}\\ {\text{PDC}}_{T,s}\left(v\right)={\text{PDC}}_{E,s}\left(v\right)\frac{G_{\text{Rx},\text{E},\text{s}}\left(v\right)}{G_{\text{Rx},\text{T},\text{s}}\left(v\right)}=\frac{P_{s}\left(v\right)}{G_{\text{Rx},\text{T},\text{s}}\left(v\right)} \end{array} \end{array}$$ (9)

Here, *G*_Rx,E_ is the stored effective relative receive divisor, whereas *G*_Rx,T_ is the leave-one-out template fitted to complete *G*_Rx,E_ fields from the other participants. *G*_Rx,E_ cancels only in the algebraic undo of the retained Arm-E PDC; a separate common ${\text{b}}_{1}^{+}$ cancellation between Arm E and Arm T is not assumed. The substitution in Equation 9 was evaluated only where the effective *G*_Rx,T_ divisor was finite and nonzero; invalid or nonpositive values were excluded through the established support and validity handling. Template coefficients and receive-field values were obtained exclusively from the other 24 participants. Deployment used the held-out participant's pre-existing *T*1/SPM-derived brain bounding box to define the spatial evaluation frame; no held-out receive field or qWC outcome was used in template fitting, normalization, or scale selection.

Each arm then independently repeated the residual-calibration chain. The local intercept C0 was estimated from the inverse-PD/inverse-*T*1 relationship and spatially regularized to give the residual calibration field μ. Its mean-normalized form used by the CSF routine is denoted as μ. A stored intermediate-PD artifact, *PD*_stored_ = clip[PDC × μ,(0,2)], was retained for diagnostic purposes but was not read by the ventricular-CSF calibration routine. Within that routine, μ was mean-normalized over finite nonzero voxels with positive PDC to obtain μ, and the true pre-CSF numerator was PDC × μ. Both μ and μ are residual calibration fields, not measured receive-sensitivity fields.

Ventricular CSF was identified independently in each arm using the harmonized study criteria 2.9 ≤ *T*1 ≤ 5.9 s and $T_{2}^{*}$ ≥ 0.5 s within the established CSF-calibration framework (Abbas et al., 2015). Transmit support was represented by 0.01-wide, lower-inclusive/upper-exclusive bins spanning 0.90 ≤ ${\text{b}}_{1}^{+}$ < 1.20; at least 11 voxels per retained bin. One CSF proton-density estimate was formed per retained bin, and the final ventricular-CSF scalar was the voxel-count-weighted mean across retained bins. Fractional quantitative proton density (Qpd) was then formed as (PDC × μ)/*PD*_CSF_ using that arm's independently estimated scalar; values outside [0,2.5] were set to zero before the stricter endpoint-validity rule was applied. The scalar was not shared between arms, and the template was never rescaled to the held-out participant, matched to Arm-E medians, or adjusted using held-out qWC outcomes.

Fractional Qpd is dimensionless. Reader-facing qWC, denoted *W* in Equation 10, is expressed in p.u.:


$$\begin{array}{l} W_{a,s}\left(v\right)=100Q_{a,s}\left(v\right) \end{array}$$ (10)

### 2.7 Tissue masks, geometry, and validity

Tissue probability maps were generated from the established quantitative *T*_1_ input using SPM25 (Ashburner and Friston, 2005). These maps defined high-confidence white matter (WM), gray matter (GM), and cerebrospinal fluid (CSF) class supports at probability ≥0.98; these maps were not qWC endpoints. WM and GM were the primary estimands, and combined brain was secondary. No new segmentation was performed, masks were not modified, and no CSF tissue-agreement endpoint was calculated.

For region *r* and participant *s, M*_*r,s*_[*i,j,k*] denotes the binary regional mask; *Q*_*a,s*_[*i,j,k*] denotes fractional Qpd in arm *a*ε{*E,T*}. Masks were applied by direct array indexing using these co-located arrays. No resampling, reorientation, interpolation, registration, or header rewriting was performed for endpoint extraction. For every participant, Arm-T and Arm-E Qpd volumes were verified to match exactly in dimensions, affine, qform and sform matrices and codes, voxel sizes, and orientation.

Arm-specific validity was defined by finiteness and strict fractional-Qpd bounds. Arm-specific validity, symmetric common-validity support, and participant-level regional endpoints are defined in Equations 11–13.


$$\begin{array}{l} V_{a,s}\left(v\right)=\{\begin{array}{cc} 1 & if\;Q_{a,s}\left(v\right)\text{ is finite and }0.3<Q_{a,s}\left(v\right)<1.2\\ 0 & \text{otherwise} \end{array} \end{array}$$ (11)

For region *r* and participant *s*, the symmetric common-validity support was defined as:


$$\begin{array}{l} C_{r,s}=\left\{v:M_{r,s}\left(v\right)=1,V_{E,s}\left(v\right)=1,V_{T,s}\left(v\right)=1\right\} \end{array}$$ (12)

In plain terms, this is the set of voxels that satisfy the same valid-qWC criteria in both arms being compared. The participant-level regional endpoint was the median qWC on that same support in each arm:


$$\begin{array}{l} m_{a,r,s}=\underset{v\in C_{r,s}}{\text{median}}(W_{a,s}(v)) \end{array}$$ (13)

If the common support was empty, the regional endpoint was undefined and the participant-region failed endpoint extraction; no participant-region had empty common support.

### 2.8 Statistical analysis

The statistical unit was the participant (*n* = 25). The comparison direction was Arm T minus Arm E. For region *r* and participant *s*, the paired difference in p.u. was the paired differences, cohort summaries, confidence intervals, agreement measures, coverage, and concordance coefficient are defined in Equations 14–21.


$$\begin{array}{l} d_{r,s}=m_{T,r,s}-m_{E,r,s} \end{array}$$ (14)

The mean and sample standard deviation of paired differences were


$$\begin{array}{l} {\bar{d}}_{r}=\frac{1}{n}\overset{n}{\underset{s=1}{\sum }}d_{r,s} \end{array}$$ (15)

$$\begin{array}{l} s_{d,r}=\sqrt{\frac{1}{n-1}\overset{n}{\underset{s=1}{\sum }}{\left(d_{r,s}-{\bar{d}}_{r}\right)}^{2}} \end{array}$$ (16)

A two-sided 95% Student-t confidence interval with 24 degrees of freedom was used:


$$\begin{array}{l} {\bar{d}}_{r}\pm t_{0.975,24}\frac{s_{d,r}}{\sqrt{n}} \end{array}$$ (17)

Mean absolute error and Bland–Altman limits of agreement were calculated (Bland and Altman, 1986):


$$\begin{array}{l} {\text{MAE}}_{r}=\frac{1}{n}\overset{n}{\underset{s=1}{\sum }}|d_{r,s}| \end{array}$$ (18)

$$\begin{array}{l} {\text{LoA}}_{r}={\bar{d}}_{r}\pm 1.96s_{d,r} \end{array}$$ (19)

Common-validity coverage was as follows:


$$\begin{array}{l} {\text{Coverage}}_{r,s}=100\frac{\left|C_{r,s}\right|}{\left|M_{r,s}\right|} \end{array}$$ (20)

Pearson *r* was reported as an association, not an agreement. The concordance correlation coefficient (CCC) used population moments (Lin, 1989):


$$\begin{array}{l} \rho_{c}=\frac{2\sigma_{ET}}{\sigma_{E}^{2}+\sigma_{T}^{2}+{\left(\mu_{E}-\mu_{T}\right)}^{2}} \end{array}$$ (21)

The CCC expression uses the Arm-E and Arm-T population means and variances and their population covariance for the paired regional medians. Confidence intervals for mean difference were descriptive; no equivalence or noninferiority analysis was performed.

### 2.9 Reproducibility and prespecification

Exact leave-one-out membership and polynomial-degree sensitivity are provided in Supplementary Tables S1, S2. The ordered cohort, arm definitions, Qpd validity bounds, tissue threshold, participant-level summary, comparison direction, and agreement statistics were fixed before inspection of the completed Arm-T cohort outcomes. The total polynomial degree of 3 was fixed as the deployed specification before Arm-T qWC outcomes were produced. A later sensitivity analysis, conducted in relative receive-field space only and using no qWC outcome, showed modestly lower held-out mismatch for degree 5 (Supplementary Table S2); degree 3 was retained unchanged and is not presented as empirically optimal. The primary symmetric ventricular-CSF calibration definition was fixed before the historical symmetric threshold sensitivity was inspected. All 25 Arm-T processing runs were completed, and endpoint extraction proceeded only after exact Arm-T/Arm-E Qpd geometry and compatibility with direct-index mask application had been verified for every participant, as described under “Tissue Masks, Geometry and Validity.”

### 2.10 Training-size sensitivity analysis

Training-size sensitivity was evaluated in relative receive-field space to characterize how receive-template fidelity varied with the number of participants contributing to template construction. For each held-out participant, the eligible training pool comprised exactly the other 24 participants. The fixed degree-3 arithmetic-mean template was evaluated at training sizes of 4, 8, 12, 16, 20, and 24 participants. For training sizes 4–20, 200 subsets were sampled from the eligible pool without replacement within each subset. A fixed deterministic pseudorandom sampling scheme was used to ensure exact reproducibility. The held-out participant was never eligible for inclusion. At *n* = 24, the complete other-24 set was unique and was evaluated once. Polynomial fitting was restricted to normalized-grid locations with finite data from at least 80% of the selected training participants. Template error was quantified as the median absolute relative mismatch on covered voxels. Peripheral/rim support was defined by Euclidean radius in the normalized [−1, 1]^3^ coordinate frame, with radius ≥ 0.75. For each participant and training size, subset-to-subset variability was summarized by the interquartile range. The 200 subsets quantified sensitivity to training-set composition and were not treated as independent biological observations. No qWC outcome was used, and no minimum qWC training size was inferred from this analysis.

### 2.11 Exploratory receive-input controls

After completion of the primary analysis, two exploratory sensitivity controls were added to assess the extent to which the downstream chain buffered receive-input error. In the no-receive control, only the effective receive divisor was set to unity (*G*_Rx_ = 1); physical ${\text{B}}_{1}^{+}$ transmit correction and all other upstream inputs were retained. In a single normalized-axis mirrored-template control, the degree-3 template fixed for the primary analysis was evaluated after the coordinate reflection x → –x. This axis was not assigned an anatomical left–right meaning. For both controls, all remaining processing—including independent C0/μ estimation, μ normalization, arm-specific ventricular-CSF calibration, Qpd/qWC validity, masks, and endpoint extraction—was repeated unchanged. Exactly one mirrored perturbation was used, and no outcome-guided perturbation selection was performed. These controls were *post-hoc* relative to the original study design but defined before their outcomes were inspected; they were exploratory sensitivity analyses, not prespecified primary comparisons.

### 2.12 Exploratory spatial and geometry analyses

To describe local Arm-T-minus-Arm-E disagreement without treating voxels as independent observations, each participant's brain-relative normalized-grid support was divided by Euclidean radius in the normalized [−1, 1]^3^ coordinate frame into central (radius < 0.75) and peripheral/rim (radius ≥ 0.75) zones. The 0.75 threshold was an operational descriptive boundary, consistent with the training-size analysis, rather than a biological boundary. Within white matter and gray matter, participant-level summaries were the median signed difference, median absolute difference, 95th percentile of absolute difference, spatial standard deviation, and valid coverage. Group maps display the cohort median signed difference, median absolute difference, and participant contribution at normalized-grid locations contributed by at least 80% of participants; they are descriptive and contain no voxelwise inference (Supplementary Table S4; Supplementary Figure S2).

An image-derived head-position/geometry proxy was defined as the Euclidean displacement of each participant's brain-mask centroid in scanner coordinates from the cohort-average centroid. Its participant-level Spearman associations with global and rim receive-template mismatch and with absolute white- and gray-matter qWC differences were summarized using 95% confidence intervals from a participant-level bootstrap with 10,000 resamples. As centroid displacement can reflect participant positioning, brain geometry, and segmentation differences simultaneously, it was treated as an image-derived geometry proxy rather than a direct measure of head motion or exact coil-relative head position (Supplementary Table S5).

## 3 Results

### 3.1 Leave-one-out template fidelity

All 25 held-out templates used exactly the other 24 participants with zero self-inclusion. Median relative mismatch between *G*_Rx,T_ and the held-out *G*_Rx,E_ averaged 2.273% (sample SD, 0.568%); the cohort median was 2.234%, and the range was 1.431%−3.615% (Figures 2, 3). Representative effective receive-field, template, and qWC maps illustrate the substitution (Figure 2). Residual mismatch was not spatially uniform; it was largest toward the brain periphery (Figure 2iii). This independently normalized relative metric does not, by itself, establish qWC agreement.

**Figure 2:**
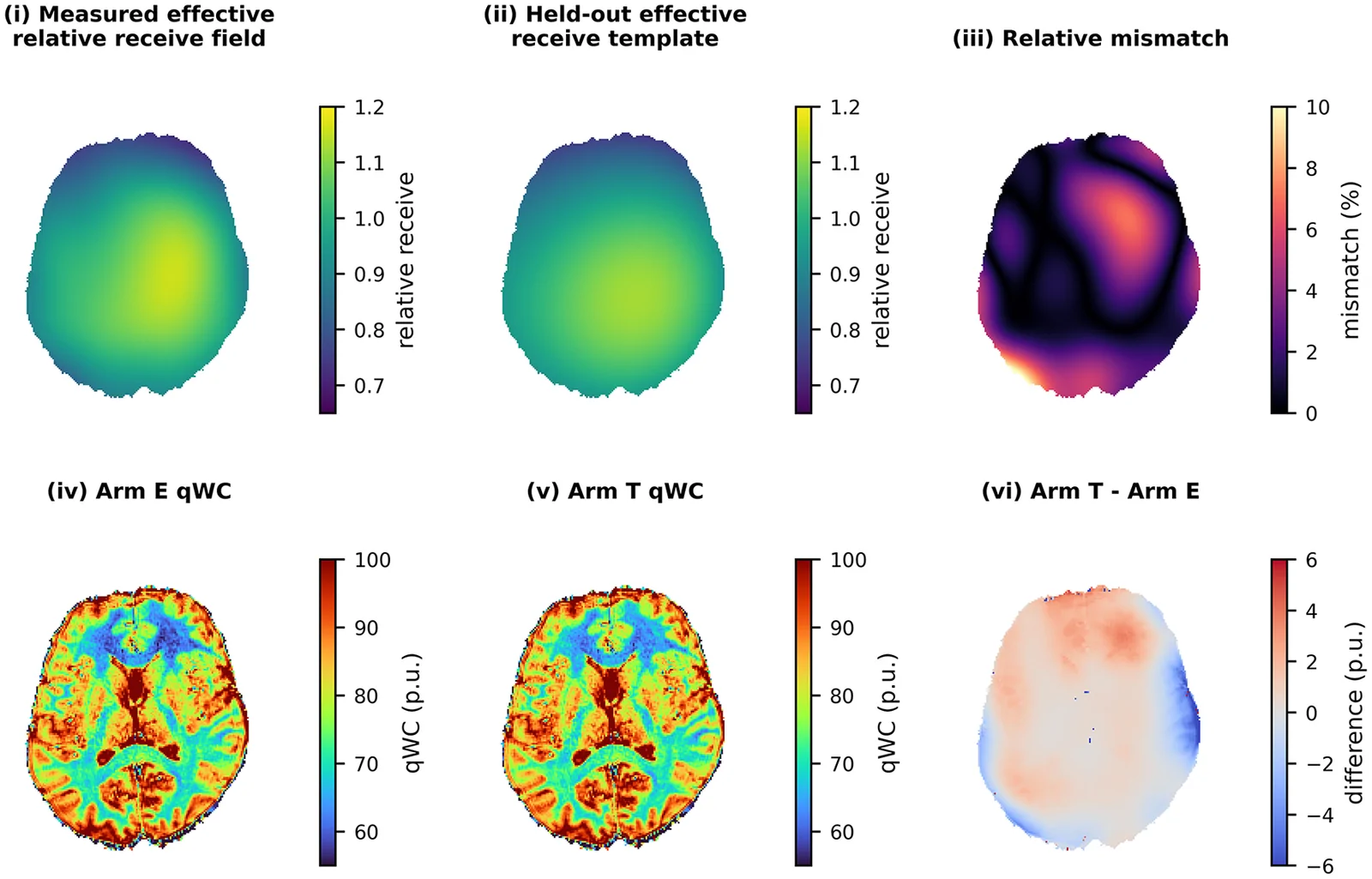
Representative held-out receive-template application (Participant-1; display slice 44). **(i)** Independently normalized measured effective relative receive field; **(ii)** the template constructed from exactly the other 24 participants; **(iii)** voxelwise relative mismatch; **(iv)** independently calibrated Arm E qWC; **(v)** independently calibrated Arm T qWC; and **(vi)** Arm T minus Arm E difference. Both qWC panels use the primary symmetric ventricular-CSF definition. Relative-receive panels share an identical scale, as do the qWC panels; qWC and differences are in p.u. Images were rotated 90° clockwise for display. A broader, morphologically closed anatomical support and nearest-valid rendered fill were used only for visual continuity; source NIfTI volumes, analysis masks, validity rules, and numerical endpoints were unchanged.

**Figure 3:**
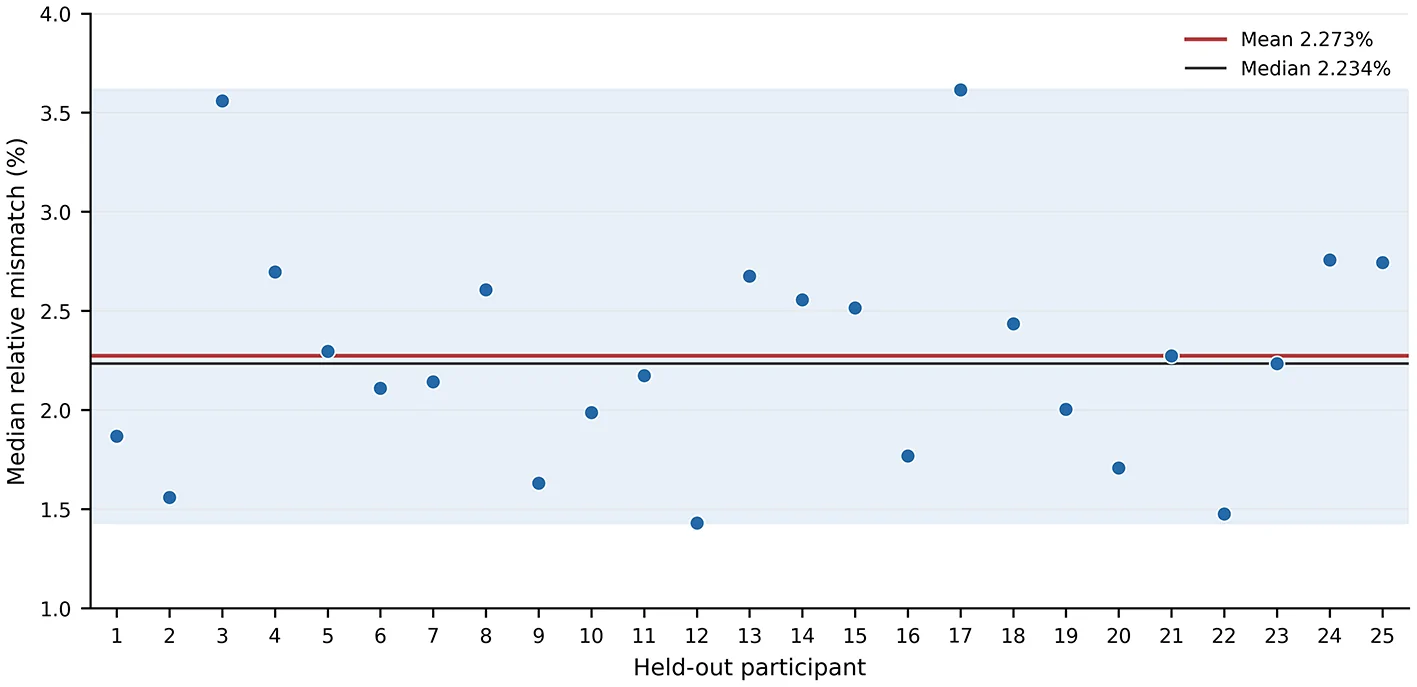
Participant-level effective relative receive-template fidelity (*n* = 25). Each point is the median relative mismatch between held-out *G*_Rx,T_ and measured *G*_Rx,E_ after independent within-brain normalization. Horizontal lines mark the mean (2.273%) and median (2.234%); shading marks the range (1.431%−3.615%). This relative field metric is not a measure of absolute qWC accuracy.

### 3.2 qWC agreement

All 25 Arm-T datasets were processed and passed exact Arm-T/Arm-E Qpd geometry verification. For white matter, mean participant-level median qWC was 69.98 p.u. in Arm E and 69.94 p.u. in Arm T. Mean paired difference was −0.04 p.u. (95% CI, −0.12 to +0.05), paired difference SD was 0.21 p.u., MAE was 0.17 p.u., and Bland–Altman limits were −0.45 to +0.37 p.u. Pearson *r* was 0.992, and Lin CCC was 0.991 (Table 2; Figures 4, 5).

**Table 2:** Primary Arm E/Arm T agreement for participant-level regional median qWC (*n* = 25).

Region	Arm E mean (p.u.)	Arm T mean (p.u.)	Bias T–E (95% CI) (p.u.)	SD (p.u.)	MAE (p.u.)	LoA (p.u.)	*r*	Lin CCC	Coverage (%)
White matter (primary)	69.98	69.94	−0.04 (−0.12 to +0.05)	0.21	0.17	−0.45 to +0.37	0.992	0.991	99.995
Gray matter (primary)	85.34	85.18	−0.17 (−0.27 to −0.07)	0.25	0.22	−0.65 to +0.31	0.993	0.989	99.48
Whole brain (secondary)	83.56	83.45	−0.12 (−0.21 to −0.02)	0.23	0.19	−0.57 to +0.33	0.994	0.993	97.17

Bias and limits are Arm T minus Arm E. SD is the sample SD of paired differences; MAE is the mean absolute paired difference; LoA are Bland–Altman limits; CCC uses population moments; coverage is the symmetric common-validity support as a percentage of the regional mask. qWC quantities and absolute differences are reported in percentage units (p.u.), defined relative to pure water (100 p.u.); r is the Pearson correlation coefficient and is not an agreement measure. Bias was calculated from the unrounded participant-level paired values and therefore may not equal the difference between the displayed rounded arm means.

**Figure 4:**
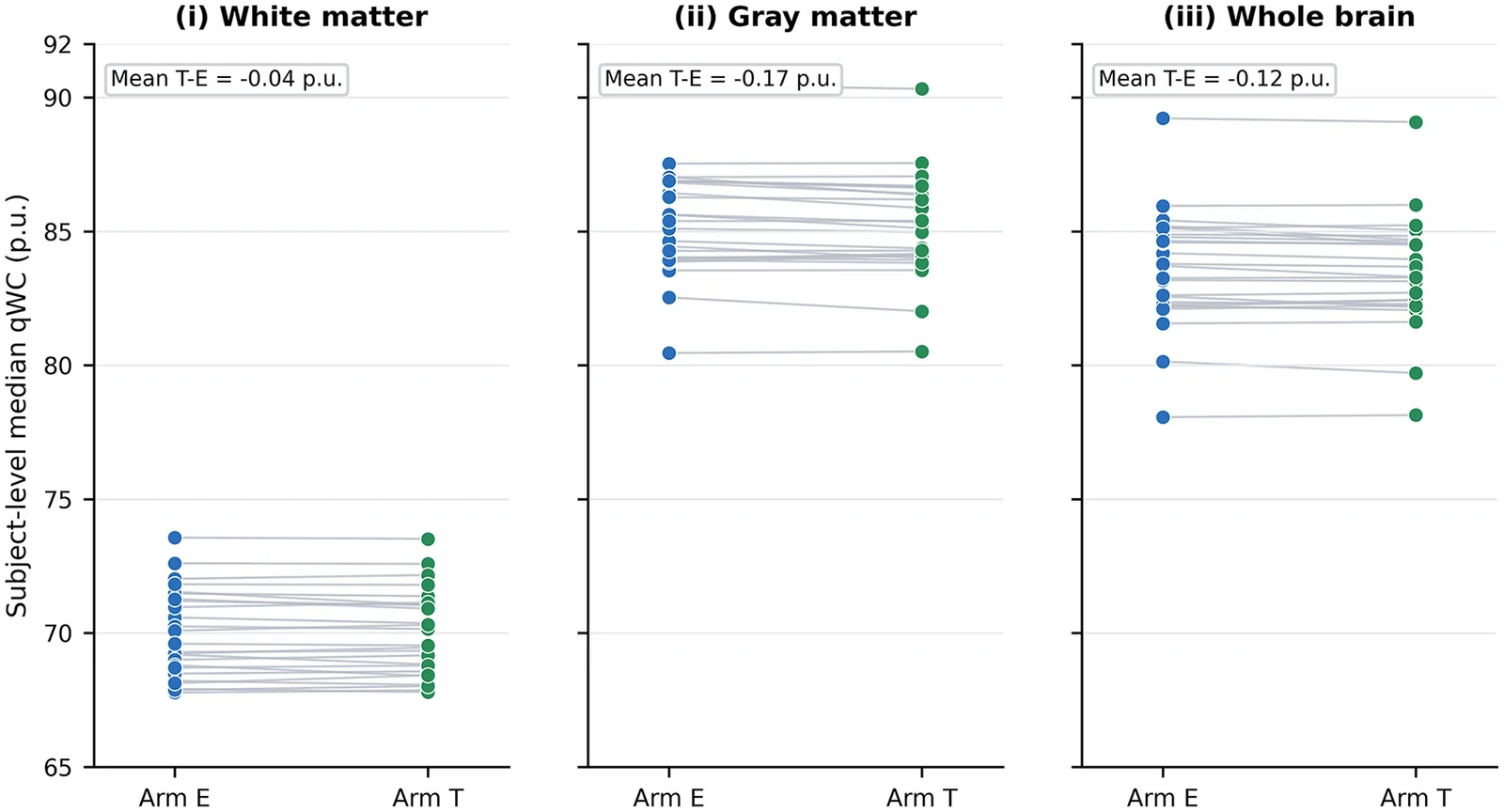
Paired participant-level regional median qWC for Arm E and Arm T (*n* = 25): **(i)** white matter, **(ii)** gray matter, and **(iii)** whole brain. Lines join values from the same participant. Regional medians use symmetric Arm-E/Arm-T common-validity support. All panels use the identical 65–92 p.u. qWC scale; annotations give the mean Arm-T-minus-Arm-E difference. All 25 complete participant pairs are visible in every panel.

**Figure 5:**
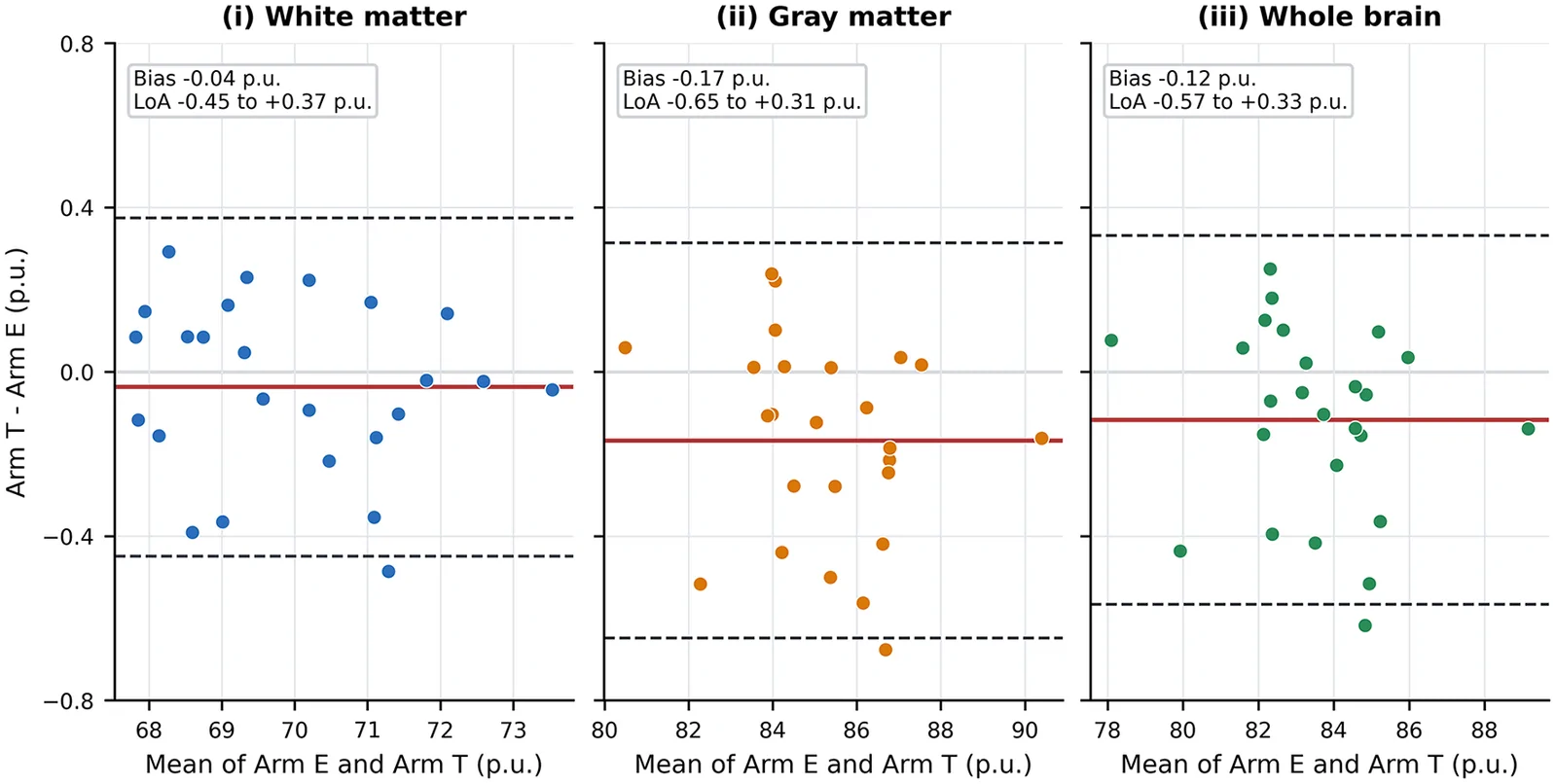
Bland–Altman plots for participant-level regional median qWC (*n* = 25): **(i)** white matter, **(ii)** gray matter, and **(iii)** whole brain. Differences are Arm T minus Arm E in p.u. Red lines show mean differences; dashed black lines show the corresponding mean ± 1.96 sample-SD limits of agreement. All panels use the identical −0.8 to +0.8 p.u. difference scale. The participant is the statistical unit, and no equivalence margin was prespecified.

For gray matter, mean participant-level median qWC was 85.34 p.u. in Arm E and 85.18 p.u. in Arm T. Mean paired difference was −0.17 p.u. (95% CI, −0.27 to −0.07), paired-difference SD was 0.25 p.u., MAE was 0.22 p.u., and limits were −0.65 to +0.31 p.u. Pearson *r* was 0.993, and Lin CCC was 0.989.

For the secondary whole-brain estimand, mean participant-level median qWC was 83.56 p.u. in Arm E and 83.45 p.u. in Arm T. The mean paired difference was −0.12 p.u. (95% CI, −0.21 to −0.02), paired-difference SD was 0.23 p.u., MAE was 0.19 p.u., and limits were −0.57 to +0.33 p.u. Pearson *r* was 0.994, and Lin CCC was 0.993. The largest absolute participant-level differences were 0.49 p.u. in white matter and 0.68 p.u. in gray matter; all prespecified participants were retained.

### 3.3 Common-validity coverage

Mean common-validity coverage was 99.995% for white matter, 99.48% for gray matter, and 97.17% for whole brain. The symmetric support ensured that each paired regional median was based on voxels valid in both arms. Every participant contributed to every prespecified endpoint; no *post-hoc* coverage threshold or participant exclusion was introduced.

### 3.4 Exploratory mechanistic and robustness analyses

No meaningful association was observed between global receive-template mismatch and participant-level qWC disagreement. Pearson *r* values for signed Arm-T-minus-Arm-E differences were −0.234 (bootstrap 95% CI, −0.548 to +0.079), +0.093 (−0.328 to +0.443), and +0.057 (−0.392 to +0.420) for white matter, gray matter, and whole brain, respectively. Corresponding values for absolute differences were +0.125 (−0.211 to +0.520), −0.102 (−0.451 to +0.337), and −0.049 (−0.381 to +0.334). All 95% participant-bootstrap confidence intervals spanned zero. With 25 participants, these exploratory analyses have limited power to detect weak associations and do not establish that none exists.

Calibration-chain decomposition showed mean Arm-T-vs.-Arm-E changes of +6.521% in participant-level median PDC, +6.202% in the pre-CSF PDC × μ numerator, and +6.349% in the independently estimated ventricular-CSF scalar (Figure 6). Thus, the approximately 6% increase in the pre-CSF quantity was accompanied by an approximately 6% increase in the arm-specific CSF-calibration scale, leaving only small residual differences in final qWC. The executed scale trace showed cohort means of the participant-level median deployed divisors of 1.076178 for *G*_Rx,E_ and 1.012541 for *G*_Rx,T_. The cohort mean and median of the participant-level *G*_Rx,T_/*G*_Rx,E_ ratios were 0.941135 and 0.938790, respectively, while the mean and median participant-level inverse-divisor shifts were +6.290% and +6.520%, respectively. These shifts were similar in magnitude to the observed PDC, pre-CSF numerator, and ventricular-CSF scalar changes, consistent with a predominantly global difference in deployed divisor scale followed by substantial compensation through arm-specific calibration. As these quantities involve different spatial operations and cohort summaries, their numerical proximity is descriptive and is not interpreted as an exact algebraic identity or as a causal partition of every residual effect. Residual median-normalized spatial mismatch in the scale-trace diagnostic was 2.259%; this diagnostic was computed on the scale-trace support rather than on the common support used for the 2.273% primary receive-field mismatch and is therefore not identical to it; the final mean whole-brain qWC difference was −0.117 p.u. (Supplementary Table S6). The results of the training-size sensitivity analysis are shown in Figure 7.

**Figure 6:**
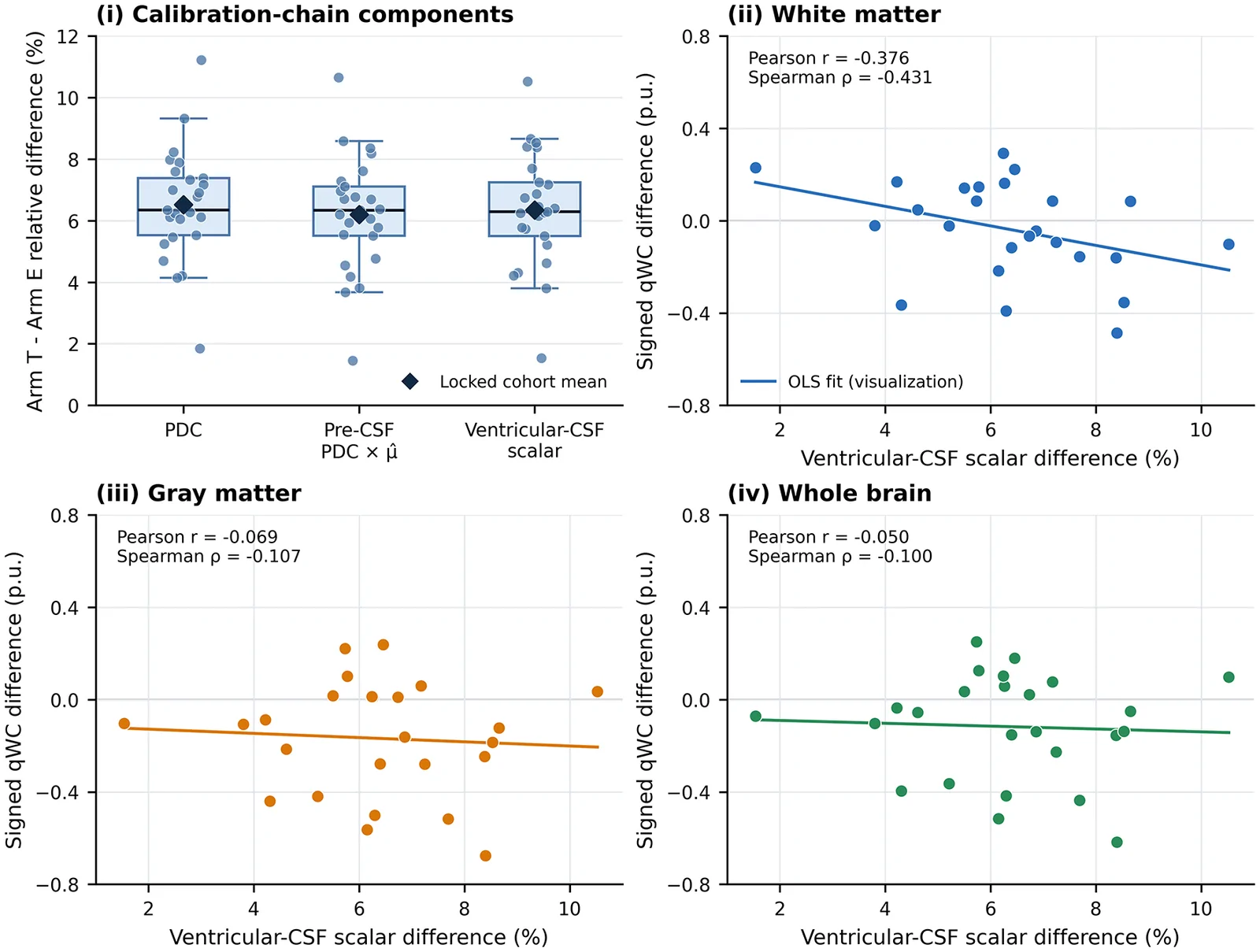
Exploratory calibration-chain decomposition. **(i)** Participant-level Arm-T-vs.-Arm-E relative differences in PDC, the pre-CSF PDC × μ numerator, and the independently estimated ventricular-CSF scalar. **(ii–iv)** Ventricular-CSF scalar difference vs. signed Arm-T-minus-Arm-E qWC difference for white matter, gray matter, and whole brain; lines are ordinary least-squares fits for visualization. The approximately 6% pre-CSF amplitude increase in Arm T was accompanied by a similar arm-specific CSF-scale increase, leaving small residual differences in final qWC. Because the arm-specific scalar enters algebraically in the qWC denominator, these associations are consistent with calibration coupling and are not statistically independent evidence that CSF calibration uniquely causes the residual qWC difference. This exploratory decomposition describes the evaluated chain and does not establish a universal mechanism.

**Figure 7:**
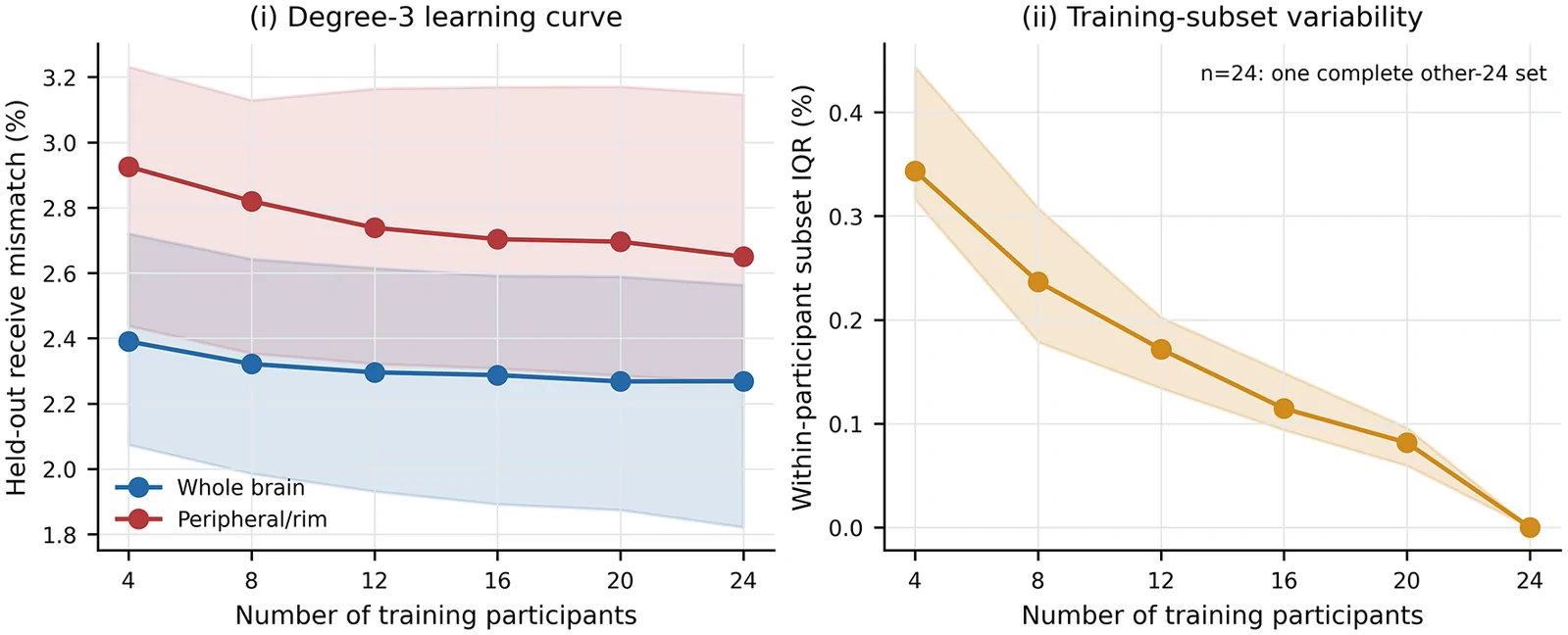
Degree-3 training-size sensitivity in relative receive-field space. **(i)** Whole-brain and peripheral/rim held-out mismatch across training sizes 4, 8, 12, 16, 20, and 24; bands show cohort interquartile ranges. **(ii)** Cohort median within-participant subset interquartile range. Two hundred subsets per held-out participant were sampled for *n* = 4–20; *n* = 24 has one complete other-24 set. This descriptive plateau does not define a minimum training size for qWC performance or establish equivalence, accuracy, or interchangeability.

Across 25 leave-one-participant-out influence runs, mean-bias ranges were −0.051 to −0.019 p.u. for white matter, −0.185 to −0.147 p.u. for gray matter, and −0.132 to −0.096 p.u. for whole brain; corresponding CCC ranges were 0.989–0.993, 0.984–0.991, and 0.990–0.994, respectively. No single participant accounted for the residual group-level differences. Coverage-vs.-absolute-difference associations and participant-level spatial summaries were descriptive and did not alter the interpretation; detailed records are available from the corresponding author on reasonable request.

When the historical ventricular-CSF criteria were applied symmetrically to both arms, mean differences remained small (white matter, −0.006 p.u.; gray matter, −0.131 p.u.; whole brain, −0.080 p.u.). The maximum absolute change from the primary analysis was 0.0368 p.u. for bias and 0.0319 p.u. for MAE.

### 3.5 Exploratory receive-input control, spatial, and geometry results

Both exploratory controls were completed for all 25 participants. Arm T had 2.273% receive mismatch and white-matter, gray-matter, and whole-brain MAE/CCC values of 0.170/0.991, 0.224/0.989, and 0.193/0.993, respectively. The no-receive control had 7.409% mismatch and corresponding MAE/CCC values of 0.850/0.808, 0.794/0.889, and 0.932/0.851. The normalized-axis mirrored template had 10.140% mismatch and MAE/CCC values of 0.547/0.900, 0.737/0.872, and 0.779/0.878 (Supplementary Table S3). Thus, both deliberately less-informative or spatially perturbed receive inputs were less favorable than Arm T, while regional MAEs remained below 1 p.u., consistent with substantial—but incomplete—buffering by downstream processing. Receive mismatch and regional qWC error were not monotonic across the controls: the mirrored template had greater mismatch but smaller regional qWC errors than the no-receive control.

Local differences exceeded the primary participant-level regional-median differences, particularly in the rim. For white matter, the cohort means of participant-level central vs. rim summaries were 0.285 vs. 0.340 p.u. for median absolute difference, 1.217 vs. 1.819 p.u. for the 95th percentile of absolute difference, and 0.538 vs. 0.780 p.u. for spatial standard deviation. For gray matter, the corresponding values were 0.337 vs. 0.539 p.u., 2.053 vs. 3.183 p.u., and 0.870 vs. 1.375 p.u., respectively. Mean valid coverage remained 99.44–100.00% (Supplementary Table S4; Supplementary Figure S2).

For the image-derived head-position/geometry proxy, Spearman ρ was 0.333 (95% bootstrap CI, −0.051 to 0.642) for global receive mismatch and 0.438 (0.085 to 0.699) for rim mismatch. Associations with absolute white- and gray-matter qWC differences were −0.229 (−0.588 to 0.213) and −0.141 (−0.535 to 0.307), respectively. The proxy showed a modest descriptive positive association with rim mismatch; intervals for global mismatch and regional qWC disagreement did not support clear associations (Supplementary Table S5).

## 4 Discussion

Using a strict leave-one-participant-out approach in 25 healthy participants, a receive template constructed from the other 24 participants reproduced each held-out effective relative receive field, with a mean mismatch of 2.273%. Under symmetric, independently estimated downstream calibration, Arm T then closely tracked Arm E: mean differences were −0.04 p.u. in white matter, −0.17 p.u. in gray matter, and −0.12 p.u. in whole brain. Receive-field fidelity and final qWC agreement answer related but distinct questions and should not be conflated.

However, mean differences alone do not characterize agreement. White- and gray-matter MAE values were 0.17 and 0.22 p.u.; Bland–Altman widths were 0.82 and 0.96 p.u.; and Lin CCC values were 0.991 and 0.989. Pearson correlations were similarly high. These results indicate close participant-level agreement and small residual differences within the evaluated configuration, but no prospective equivalence, noninferiority, or clinical acceptability margin was defined.

The results are consistent with the broader observation that smoothly varying receive and bias fields are difficult to separate from low-spatial-frequency tissue contrast when estimated from the target image itself (Vovk et al., 2007; Mezer et al., 2016). A fixed-population field avoids adapting to the held-out qWC image but assumes repeatability of the scanner, receive configuration, and subject positioning within the evaluated setting (Oros-Peusquens et al., 2019).

Global receive-template mismatch showed no meaningful association with signed or absolute qWC disagreement. Part of this dissociation follows from how the two quantities are defined: the mismatch statistic compares the two fields after each has been independently median-normalized and is therefore blind to a difference in global scale, whereas the qWC endpoint retains whatever global component survives independent ventricular-CSF calibration. A single global field-fidelity metric is therefore insufficient to predict individual downstream qWC performance, and the 2.273% receive mismatch should not be propagated directly as a bound or estimate of qWC disagreement.

The independently re-estimated C0/μ and CSF-calibration chain substantially compensated for the receive-template-induced change in precursor scale. Arm T showed approximately +6.5% PDC and +6.2% pre-CSF numerator relative to Arm E, while its independently estimated ventricular-CSF scalar was approximately +6.35%. These similar multiplicative changes left only small residual qWC differences (Figure 6). The processing chain supports this compensation interpretation, but it does not establish that the same behavior will occur under other scanners, receive configurations, or participant-positioning conditions. The closely similar result obtained with the historical symmetric CSF definition further indicates descriptive threshold robustness rather than formal equivalence. As the independently estimated ventricular-CSF scalar enters algebraically in the final qWC denominator, its association with Arm-T-minus-Arm-E qWC differences is expected to some extent and is mechanistically consistent with calibration coupling; it is not statistically independent evidence that CSF calibration uniquely causes the residual difference.

The exploratory receive-input controls refine this interpretation. Both the unit-divisor no-receive control and the single spatially mirrored template produced larger receive mismatch and less favorable regional qWC agreement than the correctly constructed template. The downstream pipeline was therefore not completely insensitive to receive information, and the template retained spatial receive information relevant to final qWC. Nevertheless, receive mismatches of approximately 7.4–10.1% were accompanied by regional qWC MAEs below 1 p.u., consistent with substantial but incomplete buffering of receive-input error by residual C0/μ and CSF calibration. Regional qWC agreement alone therefore does not validate receive-field fidelity. Within these exploratory controls, the template nevertheless added measurable value: compared with the no-receive control, it reduced regional MAE approximately five-fold in white matter (0.850 vs. 0.170 p.u.) and whole brain (0.932 vs. 0.193 p.u.) and approximately 3.5-fold in gray matter (0.794 vs. 0.224 p.u.), and increased CCC from 0.808–0.889 to 0.989–0.993. The no-receive MAEs were also larger than the external tissue-mean scan–rescan standard deviations discussed below, although MAE and across-scan SD are different metrics obtained from different experimental frameworks. Receive-field mismatch and downstream qWC disagreement were not monotonic across the two controls: the mirrored template had greater receive mismatch than the no-receive control but somewhat smaller regional qWC errors. The mismatch metric is computed after independent median normalization and summarizes relative spatial-field discrepancy rather than global scale, whereas final qWC additionally depends on the spatial structure of the receive error and subsequent arm-specific residual-field and CSF calibration. The controls therefore represent qualitatively different receive-error conditions but not points on a single dose-response curve.

The spatial analysis further showed that participant-level regional medians conceal larger local positive and negative differences. Disagreement was greater toward the brain periphery, most clearly in gray matter, where the rim median absolute difference was 0.539 p.u. and the 95th-percentile absolute difference was 3.183 p.u. These descriptive findings support cautious regional-level interpretation only within the evaluated setting and argue against interpreting the small regional-median biases as evidence of spatial interchangeability. Because the rim 95th-percentile absolute differences (1.819 p.u. in white matter and 3.183 p.u. in gray matter) approach the few-percentage-point changes that can accompany pathology, the present results do not establish the suitability of the template for voxel- or lesion-level qWC measurement, particularly in cortical or peripheral regions.

The brain-relative coordinate construction is both an enabling assumption and a limitation. Bounding-box normalization allows approximately corresponding brain-relative locations to be pooled, but normalizes away absolute translation and physical scale relative to the receive array and does not explicitly model coil-relative head position. The exploratory image-derived geometry proxy showed a modest association with rim mismatch, while uncertainty remained broad for global mismatch and regional qWC differences. It was not a direct measure of head motion or exact coil-relative head position. Future studies should retain or explicitly model scanner/coil coordinates and prospectively vary participant position.

The intended use case is a previously established scanner/receive-coil configuration in which fixed template coefficients can subsequently be deployed in otherwise compatible datasets without participant-specific receive-field fitting or rescaling and without acquiring the individual matched PA/BC reference pair. This removes approximately 1.1 min of participant-specific receive-reference acquisition and its associated receive-field derivation, which may be useful for retrospective datasets, large cohorts, and time-constrained research or clinical workflows. The trade-off is configuration specificity, lack of external scanner/coil validation, no explicit participant-specific coil-relative positioning, persistent local/rim differences, and dependence of the observed final robustness on downstream calibration.

Same-pipeline scan-rescan repeatability was not measured in this 25-participant study. As external context only, Oros-Peusquens et al. reported tissue-mean across-scan standard deviations of 0.33 p.u. in white matter and 0.38 p.u. in gray matter in one 23-year-old volunteer scanned 12 times across three sessions with complete repositioning and shimming on a 3 T Tim-TRIO with a 12-channel receive coil and a different qWC protocol; whole-brain voxelwise standard deviation had a mean of 1.02 p.u., a mode of 0.61 p.u., and values of approximately 2–4 p.u. in susceptibility-affected regions. The present Arm-T/Arm-E regional biases and MAEs were of the same order as or smaller than those external tissue-mean standard deviations. However, these between-arm agreement metrics are not equivalent to across-scan standard deviation, and the external values do not represent repeatability of the present pipeline or define clinical negligibility. Here, p.u. denotes absolute percentage units on the qWC scale, not relative percent change.

Arm E is a body-coil-referenced effective relative receive strategy and should not be interpreted as ground truth. It provides a participant-specific comparator within the established qWC framework; the small residual Arm-T differences are therefore agreement results relative to Arm E, not measures of accuracy against known biological water content (Mezer et al., 2013; Abbas et al., 2014, 2015).

Unlike protocols that jointly estimate proton density, *T*_1_, or multiple tissue properties, this study tests only receive-correction substitution within an inherited two-point estimation framework. The inherited two-point *T*_1_ estimation remains upstream of the arm substitution and was not a primary study endpoint here. Comparisons with QRAGE (Zimmermann et al., 2025), MR fingerprinting (Ma et al., 2013), EPTI (Wang et al., 2019), newer multi-protocol water-fraction work (Mama et al., 2026), and established quantitative water content methods (Neeb et al., 2006, 2008) provide context but do not establish performance equivalence across methods.

Several limitations are important. This study was based on an evaluation of 25 male participants at one site, on one specific 3 T scanner, with one specific receive configuration; it was not an independent deployment cohort. As already mentioned, Arm E serves as an explicit relative reference rather than a biological ground truth, and no prospective equivalence margin was prespecified. The configuration-specific degree-3 template was fixed prospectively. A later sensitivity analysis in relative receive-field space, with no qWC outcome, showed modestly lower held-out mismatch for degree 5, so degree 3 is not claimed to be empirically optimal. External transfer across scanners, coils, vendors, or field strengths was not tested, and generalization to repositioning or different subject loading remains uncertain. Global receive mismatch cannot be assumed to predict individual qWC disagreement, high-confidence SPM25 masks do not define a CSF agreement endpoint, and spoiled-GRE *T*1 estimates can depend on radiofrequency spoiling (Preibisch and Deichmann, 2009). The exploratory receive-input controls were not prespecified primary comparisons and cannot establish equivalence or accuracy. Local and rim differences were larger than participant-level regional-median differences, and same-pipeline repeatability was not measured.

Future work should evaluate prospectively acquired external cohorts, participant repositioning, independently varied receive configurations, and more diverse populations while retaining independent calibration and symmetric validity rules. Any clinically meaningful agreement margin should be defined before outcome inspection. The present evidence supports technical feasibility under the evaluated configuration, not universal replacement of a participant-specific receive reference.

## 5 Conclusion

In this internal 25-participant leave-one-out evaluation, a configuration-specific inter-subject effective receive template produced close participant-level regional white- and gray-matter qWC agreement with the participant-specific explicit-reference comparator under the evaluated scanner and receive configuration. The correctly constructed template produced more favorable regional qWC agreement than either the unit-divisor no-receive control or the spatially mirrored-template control, while both controls showed that downstream calibration substantially but incompletely buffered receive-input error. Local disagreement, particularly in peripheral/rim regions, exceeded the participant-level regional-median differences. External scanner/coil transfer and same-pipeline scan–rescan repeatability remain untested; the findings do not establish accuracy, equivalence, interchangeability, or general replacement of participant-specific receive referencing.

## Data Availability

The data analyzed in this study is subject to the following licenses/restrictions: access to the underlying data is restricted by institutional data-protection requirements and by the terms of the ethics approval under which the data were acquired. The datasets consist of de-identified derived quantitative human brain maps and cannot be shared publicly. De-identified derived maps, the analysis and provenance code, and the final result tables underlying all figures and tables are available from the corresponding author upon reasonable request, subject to institutional ethics approval and a data-transfer agreement. Requests to access these datasets should be directed to Zaheer Abbas, z.abbas@fz-juelich.de.
