# Supplementary material for: Inter-subject receive-template substitution for quantitative brain water content mapping at 3 T: a leave-one-out evaluation
Source: Front Neurosci. 2026 Oct 19;20:1986813. doi: 10.3389/fnins.2026.1986813 (PMC13724431; doi:10.3389/fnins.2026.1986813)
Supplement: Supplementary file 1 [file Supplementary_file_1.DOCX]

Supplementary Material

This Supplementary Material preserves the two tabulated records submitted with the original evaluation—exact 24-participant leave-one-out membership and polynomial-degree sensitivity in relative receive-field space—and adds exploratory control, spatial, scale, and geometry summaries. The fixed cohort is denoted sequentially as Participant-1 through Participant-25.

# 1 Supplementary Table S1. Exact leave-one-out membership. Ordered 24-participant membership and source provenance for all 25 holdouts.

| **Held-out participant** | **Absent** | ***n*** | **Ordered training participants** | **Degree** | **Grid** |
| --- | --- | --- | --- | --- | --- |
| Participant-1 | True | 24 | Participant-2; Participant-3; Participant-4; Participant-5; Participant-6; Participant-7; Participant-8; Participant-9 Participant-10; Participant-11; Participant-12; Participant-13; Participant-14; Participant-15; Participant-16; Participant-17 Participant-18; Participant-19; Participant-20; Participant-21; Participant-22; Participant-23; Participant-24; Participant-25 | 3 | 48x48x32 |
| Participant-2 | True | 24 | Participant-1; Participant-3; Participant-4; Participant-5; Participant-6; Participant-7; Participant-8; Participant-9 Participant-10; Participant-11; Participant-12; Participant-13; Participant-14; Participant-15; Participant-16; Participant-17 Participant-18; Participant-19; Participant-20; Participant-21; Participant-22; Participant-23; Participant-24; Participant-25 | 3 | 48x48x32 |
| Participant-3 | True | 24 | Participant-1; Participant-2; Participant-4; Participant-5; Participant-6; Participant-7; Participant-8; Participant-9 Participant-10; Participant-11; Participant-12; Participant-13; Participant-14; Participant-15; Participant-16; Participant-17 Participant-18; Participant-19; Participant-20; Participant-21; Participant-22; Participant-23; Participant-24; Participant-25 | 3 | 48x48x32 |
| Participant-4 | True | 24 | Participant-1; Participant-2; Participant-3; Participant-5; Participant-6; Participant-7; Participant-8; Participant-9 Participant-10; Participant-11; Participant-12; Participant-13; Participant-14; Participant-15; Participant-16; Participant-17 Participant-18; Participant-19; Participant-20; Participant-21; Participant-22; Participant-23; Participant-24; Participant-25 | 3 | 48x48x32 |
| Participant-5 | True | 24 | Participant-1; Participant-2; Participant-3; Participant-4; Participant-6; Participant-7; Participant-8; Participant-9 Participant-10; Participant-11; Participant-12; Participant-13; Participant-14; Participant-15; Participant-16; Participant-17 Participant-18; Participant-19; Participant-20; Participant-21; Participant-22; Participant-23; Participant-24; Participant-25 | 3 | 48x48x32 |
| Participant-6 | True | 24 | Participant-1; Participant-2; Participant-3; Participant-4; Participant-5; Participant-7; Participant-8; Participant-9 Participant-10; Participant-11; Participant-12; Participant-13; Participant-14; Participant-15; Participant-16; Participant-17 Participant-18; Participant-19; Participant-20; Participant-21; Participant-22; Participant-23; Participant-24; Participant-25 | 3 | 48x48x32 |
| Participant-7 | True | 24 | Participant-1; Participant-2; Participant-3; Participant-4; Participant-5; Participant-6; Participant-8; Participant-9 Participant-10; Participant-11; Participant-12; Participant-13; Participant-14; Participant-15; Participant-16; Participant-17 Participant-18; Participant-19; Participant-20; Participant-21; Participant-22; Participant-23; Participant-24; Participant-25 | 3 | 48x48x32 |
| Participant-8 | True | 24 | Participant-1; Participant-2; Participant-3; Participant-4; Participant-5; Participant-6; Participant-7; Participant-9 Participant-10; Participant-11; Participant-12; Participant-13; Participant-14; Participant-15; Participant-16; Participant-17 Participant-18; Participant-19; Participant-20; Participant-21; Participant-22; Participant-23; Participant-24; Participant-25 | 3 | 48x48x32 |
| Participant-9 | True | 24 | Participant-1; Participant-2; Participant-3; Participant-4; Participant-5; Participant-6; Participant-7; Participant-8 Participant-10; Participant-11; Participant-12; Participant-13; Participant-14; Participant-15; Participant-16; Participant-17 Participant-18; Participant-19; Participant-20; Participant-21; Participant-22; Participant-23; Participant-24; Participant-25 | 3 | 48x48x32 |
| Participant-10 | True | 24 | Participant-1; Participant-2; Participant-3; Participant-4; Participant-5; Participant-6; Participant-7; Participant-8 Participant-9; Participant-11; Participant-12; Participant-13; Participant-14; Participant-15; Participant-16; Participant-17 Participant-18; Participant-19; Participant-20; Participant-21; Participant-22; Participant-23; Participant-24; Participant-25 | 3 | 48x48x32 |
| Participant-11 | True | 24 | Participant-1; Participant-2; Participant-3; Participant-4; Participant-5; Participant-6; Participant-7; Participant-8 Participant-9; Participant-10; Participant-12; Participant-13; Participant-14; Participant-15; Participant-16; Participant-17 Participant-18; Participant-19; Participant-20; Participant-21; Participant-22; Participant-23; Participant-24; Participant-25 | 3 | 48x48x32 |
| Participant-12 | True | 24 | Participant-1; Participant-2; Participant-3; Participant-4; Participant-5; Participant-6; Participant-7; Participant-8 Participant-9; Participant-10; Participant-11; Participant-13; Participant-14; Participant-15; Participant-16; Participant-17 Participant-18; Participant-19; Participant-20; Participant-21; Participant-22; Participant-23; Participant-24; Participant-25 | 3 | 48x48x32 |
| Participant-13 | True | 24 | Participant-1; Participant-2; Participant-3; Participant-4; Participant-5; Participant-6; Participant-7; Participant-8 Participant-9; Participant-10; Participant-11; Participant-12; Participant-14; Participant-15; Participant-16; Participant-17 Participant-18; Participant-19; Participant-20; Participant-21; Participant-22; Participant-23; Participant-24; Participant-25 | 3 | 48x48x32 |
| Participant-14 | True | 24 | Participant-1; Participant-2; Participant-3; Participant-4; Participant-5; Participant-6; Participant-7; Participant-8 Participant-9; Participant-10; Participant-11; Participant-12; Participant-13; Participant-15; Participant-16; Participant-17 Participant-18; Participant-19; Participant-20; Participant-21; Participant-22; Participant-23; Participant-24; Participant-25 | 3 | 48x48x32 |
| Participant-15 | True | 24 | Participant-1; Participant-2; Participant-3; Participant-4; Participant-5; Participant-6; Participant-7; Participant-8 Participant-9; Participant-10; Participant-11; Participant-12; Participant-13; Participant-14; Participant-16; Participant-17 Participant-18; Participant-19; Participant-20; Participant-21; Participant-22; Participant-23; Participant-24; Participant-25 | 3 | 48x48x32 |
| Participant-16 | True | 24 | Participant-1; Participant-2; Participant-3; Participant-4; Participant-5; Participant-6; Participant-7; Participant-8 Participant-9; Participant-10; Participant-11; Participant-12; Participant-13; Participant-14; Participant-15; Participant-17 Participant-18; Participant-19; Participant-20; Participant-21; Participant-22; Participant-23; Participant-24; Participant-25 | 3 | 48x48x32 |
| Participant-17 | True | 24 | Participant-1; Participant-2; Participant-3; Participant-4; Participant-5; Participant-6; Participant-7; Participant-8 Participant-9; Participant-10; Participant-11; Participant-12; Participant-13; Participant-14; Participant-15; Participant-16 Participant-18; Participant-19; Participant-20; Participant-21; Participant-22; Participant-23; Participant-24; Participant-25 | 3 | 48x48x32 |
| Participant-18 | True | 24 | Participant-1; Participant-2; Participant-3; Participant-4; Participant-5; Participant-6; Participant-7; Participant-8 Participant-9; Participant-10; Participant-11; Participant-12; Participant-13; Participant-14; Participant-15; Participant-16 Participant-17; Participant-19; Participant-20; Participant-21; Participant-22; Participant-23; Participant-24; Participant-25 | 3 | 48x48x32 |
| Participant-19 | True | 24 | Participant-1; Participant-2; Participant-3; Participant-4; Participant-5; Participant-6; Participant-7; Participant-8 Participant-9; Participant-10; Participant-11; Participant-12; Participant-13; Participant-14; Participant-15; Participant-16 Participant-17; Participant-18; Participant-20; Participant-21; Participant-22; Participant-23; Participant-24; Participant-25 | 3 | 48x48x32 |
| Participant-20 | True | 24 | Participant-1; Participant-2; Participant-3; Participant-4; Participant-5; Participant-6; Participant-7; Participant-8 Participant-9; Participant-10; Participant-11; Participant-12; Participant-13; Participant-14; Participant-15; Participant-16 Participant-17; Participant-18; Participant-19; Participant-21; Participant-22; Participant-23; Participant-24; Participant-25 | 3 | 48x48x32 |
| Participant-21 | True | 24 | Participant-1; Participant-2; Participant-3; Participant-4; Participant-5; Participant-6; Participant-7; Participant-8 Participant-9; Participant-10; Participant-11; Participant-12; Participant-13; Participant-14; Participant-15; Participant-16 Participant-17; Participant-18; Participant-19; Participant-20; Participant-22; Participant-23; Participant-24; Participant-25 | 3 | 48x48x32 |
| Participant-22 | True | 24 | Participant-1; Participant-2; Participant-3; Participant-4; Participant-5; Participant-6; Participant-7; Participant-8 Participant-9; Participant-10; Participant-11; Participant-12; Participant-13; Participant-14; Participant-15; Participant-16 Participant-17; Participant-18; Participant-19; Participant-20; Participant-21; Participant-23; Participant-24; Participant-25 | 3 | 48x48x32 |
| Participant-23 | True | 24 | Participant-1; Participant-2; Participant-3; Participant-4; Participant-5; Participant-6; Participant-7; Participant-8 Participant-9; Participant-10; Participant-11; Participant-12; Participant-13; Participant-14; Participant-15; Participant-16 Participant-17; Participant-18; Participant-19; Participant-20; Participant-21; Participant-22; Participant-24; Participant-25 | 3 | 48x48x32 |
| Participant-24 | True | 24 | Participant-1; Participant-2; Participant-3; Participant-4; Participant-5; Participant-6; Participant-7; Participant-8 Participant-9; Participant-10; Participant-11; Participant-12; Participant-13; Participant-14; Participant-15; Participant-16 Participant-17; Participant-18; Participant-19; Participant-20; Participant-21; Participant-22; Participant-23; Participant-25 | 3 | 48x48x32 |
| Participant-25 | True | 24 | Participant-1; Participant-2; Participant-3; Participant-4; Participant-5; Participant-6; Participant-7; Participant-8 Participant-9; Participant-10; Participant-11; Participant-12; Participant-13; Participant-14; Participant-15; Participant-16 Participant-17; Participant-18; Participant-19; Participant-20; Participant-21; Participant-22; Participant-23; Participant-24 | 3 | 48x48x32 |

**Note:** Membership was fixed before Arm-T outcome inspection.

# 2 Supplementary Table S2. Polynomial-degree sensitivity — relative receive-field space only. Nested degree comparison of held-out receive-field mismatch.

| **Participant** | **Selected degree** | **Inner d2** | **Inner d3** | **Inner d4** | **Inner d5** | **Nested whole (%)** | **Fixed d3 whole (%)** | **Nested−fixed (%)** | **Nested rim (%)** | **Fixed d3 rim (%)** |
| --- | --- | --- | --- | --- | --- | --- | --- | --- | --- | --- |
| Participant-1 | 5 | 2.5947 | 2.3 | 2.2234 | 2.0918 | 1.9072 | 1.8212 | 0.086 | 1.9186 | 1.7333 |
| Participant-2 | 5 | 2.6211 | 2.3084 | 2.252 | 2.1258 | 1.2142 | 1.5572 | −0.3431 | 1.486 | 1.7394 |
| Participant-3 | 5 | 2.5073 | 2.1966 | 2.1346 | 2.0104 | 3.8556 | 4.159 | −0.3034 | 4.9765 | 5.1941 |
| Participant-4 | 5 | 2.561 | 2.2528 | 2.1891 | 2.0572 | 2.7126 | 2.7001 | 0.0125 | 3.4576 | 3.4553 |
| Participant-5 | 5 | 2.5851 | 2.2729 | 2.2114 | 2.0801 | 2.3398 | 2.2859 | 0.0539 | 2.6143 | 2.6502 |
| Participant-6 | 5 | 2.6206 | 2.2836 | 2.2283 | 2.0978 | 1.9101 | 2.078 | −0.1679 | 2.0495 | 2.2645 |
| Participant-7 | 5 | 2.5964 | 2.2896 | 2.227 | 2.1005 | 1.8341 | 2.0208 | −0.1866 | 2.8809 | 3.2525 |
| Participant-8 | 5 | 2.5859 | 2.2716 | 2.2084 | 2.0743 | 2.518 | 2.5628 | −0.0448 | 2.8373 | 2.9589 |
| Participant-9 | 5 | 2.6137 | 2.3041 | 2.2437 | 2.1152 | 1.4866 | 1.6684 | −0.1818 | 1.6429 | 1.9597 |
| Participant-10 | 5 | 2.5929 | 2.2909 | 2.2319 | 2.0977 | 1.7639 | 1.9416 | −0.1776 | 2.1128 | 2.2825 |
| Participant-11 | 5 | 2.6209 | 2.2856 | 2.2286 | 2.1011 | 1.7779 | 2.1531 | −0.3752 | 2.2755 | 2.8667 |
| Participant-12 | 5 | 2.6217 | 2.3129 | 2.25 | 2.1262 | 1.1032 | 1.3886 | −0.2854 | 1.2501 | 1.5268 |
| Participant-13 | 5 | 2.6005 | 2.2711 | 2.2114 | 2.0809 | 2.3452 | 2.662 | −0.3168 | 2.3382 | 2.8278 |
| Participant-14 | 5 | 2.5864 | 2.269 | 2.2193 | 2.0887 | 2.1408 | 2.5552 | −0.4143 | 2.4456 | 3.0057 |
| Participant-15 | 5 | 2.5796 | 2.2767 | 2.2071 | 2.075 | 2.4793 | 2.3843 | 0.095 | 3.7596 | 3.6187 |
| Participant-16 | 5 | 2.6105 | 2.2995 | 2.241 | 2.1116 | 1.469 | 1.7742 | −0.3052 | 2.0239 | 2.3233 |
| Participant-17 | 5 | 2.5551 | 2.2351 | 2.1732 | 2.0405 | 3.3982 | 3.4164 | −0.0182 | 4.3872 | 4.0768 |
| Participant-18 | 5 | 2.5952 | 2.2719 | 2.1966 | 2.0684 | 2.5336 | 2.4528 | 0.0808 | 3.0956 | 3.1458 |
| Participant-19 | 5 | 2.6197 | 2.2946 | 2.2361 | 2.1032 | 1.7284 | 2.0513 | −0.3229 | 2.4173 | 2.611 |
| Participant-20 | 5 | 2.6176 | 2.2981 | 2.2453 | 2.1107 | 1.5354 | 1.7833 | −0.2479 | 1.4859 | 1.9431 |
| Participant-21 | 5 | 2.5974 | 2.2826 | 2.2258 | 2.0966 | 2.0339 | 2.2686 | −0.2346 | 2.1247 | 2.4597 |
| Participant-22 | 5 | 2.6289 | 2.3125 | 2.2516 | 2.1259 | 1.2426 | 1.5231 | −0.2805 | 1.5815 | 1.9232 |
| Participant-23 | 5 | 2.5967 | 2.2857 | 2.2259 | 2.0967 | 1.9485 | 2.2871 | −0.3386 | 1.9648 | 2.264 |
| Participant-24 | 5 | 2.5774 | 2.2607 | 2.2079 | 2.0803 | 2.406 | 2.762 | −0.3561 | 2.5859 | 3.0093 |
| Participant-25 | 5 | 2.5695 | 2.2598 | 2.1977 | 2.0715 | 2.4155 | 2.6129 | −0.1974 | 3.0974 | 3.2725 |

**Note:** For each outer held-out participant, the other 24 formed the outer training set. An inner 24-fold leave-one-out comparison evaluated degrees 2–5 using receive-field mismatch only; the degree that minimized the mean inner transfer error was selected, with ties assigned to the smaller degree. That degree was refitted to all 24 outer-training participants and evaluated once on the outer holdout; no qWC outcome was used. The nested model had a median mismatch of 1.9485%, a worst mismatch of 3.8556%, and a mean mismatch of 2.083984% (95% CI, 1.81635–2.35162). Fixed degree 3 had a median mismatch of 2.2686%, a worst mismatch of 4.1590%, and a mean mismatch of 2.274796% (95% CI, 2.02269–2.52690). The paired nested-minus-fixed mean was −0.190812% (95% CI, −0.25731 to −0.12431), with a median of −0.2347% (*n* = 25). This later relative-field-only analysis modestly favored degree 5; the deployed degree-3 qWC analysis was not changed. These prespecified degree-3 values belong to the separate nested polynomial-degree sensitivity analysis in relative receive-field space and should not be interpreted as reproducing the primary Figure 3 mismatch summary.

# 3 Supplementary Table S3. Exploratory receive-input control agreement relative to Arm E.

All values are participant-level summaries (n = 25). Controls were post hoc sensitivity analyses added after completion of the primary analysis. Bias and limits are control minus Arm E. qWC differences are in percentage units (p.u.); receive mismatch is relative percent. B₁⁺ transmit correction and all downstream processing were retained. Exactly one normalized-axis mirrored perturbation (x → −x) was used.

| **Control** | **Region** | **Mismatch (%)** | **Bias (95% CI), p.u.** | **SD** | **MAE** | **LoA, p.u.** | **Pearson r** | **CCC** | **Coverage (%)** | **Max \|Δ\|** |
| --- | --- | --- | --- | --- | --- | --- | --- | --- | --- | --- |
| No receive | WM | 7.409 | +0.688 (+0.357 to +1.020) | 0.804 | 0.850 | −0.887 to +2.264 | 0.881 | 0.808 | 99.995 | 2.079 |
| No receive | GM | 7.409 | −0.579 (−0.907 to −0.250) | 0.796 | 0.794 | −2.138 to +0.981 | 0.927 | 0.889 | 99.475 | 2.623 |
| No receive | Whole brain | 7.409 | −0.828 (−1.201 to −0.454) | 0.905 | 0.932 | −2.601 to +0.946 | 0.915 | 0.851 | 97.147 | 3.814 |
| Mirrored template | WM | 10.140 | −0.337 (−0.613 to −0.062) | 0.668 | 0.547 | −1.647 to +0.972 | 0.920 | 0.900 | 99.995 | 1.760 |
| Mirrored template | GM | 10.140 | −0.572 (−0.899 to −0.244) | 0.793 | 0.737 | −2.126 to +0.983 | 0.918 | 0.872 | 99.475 | 2.254 |
| Mirrored template | Whole brain | 10.140 | −0.654 (−0.983 to −0.325) | 0.796 | 0.779 | −2.215 to +0.907 | 0.929 | 0.878 | 97.050 | 2.292 |

# 4 Supplementary Table S4. Center-versus-rim Arm-T minus Arm-E qWC differences.

Zones were defined in the existing brain-relative normalized frame: central radius < 0.75 and rim radius ≥ 0.75. Entries are means of 25 participant-level summaries; voxels were not treated as independent observations and no voxelwise inference was performed.

| **Tissue** | **Zone** | **Median signed Δ (p.u.)** | **Median \|Δ\| (p.u.)** | **95th percentile \|Δ\| (p.u.)** | **Spatial SD (p.u.)** | **Coverage (%)** |
| --- | --- | --- | --- | --- | --- | --- |
| WM | central | −0.043 | 0.285 | 1.217 | 0.538 | 99.999 |
| WM | rim | −0.036 | 0.340 | 1.819 | 0.780 | 99.982 |
| GM | central | −0.095 | 0.337 | 2.053 | 0.870 | 99.530 |
| GM | rim | −0.155 | 0.539 | 3.183 | 1.375 | 99.439 |

# 5 Supplementary Table S5. Image-derived head-position/geometry proxy associations.

The proxy was the scanner-space displacement of each participant’s brain-mask centroid from the cohort-average centroid. Spearman associations use the participant as the unit and 10,000 bootstrap replicates. The proxy is descriptive and is not a measure of head motion.

| **Outcome** | **Spearman ρ** | **Bootstrap 95% CI** | **n** |
| --- | --- | --- | --- |
| Global receive mismatch | 0.333 | −0.051 to 0.642 | 25 |
| Rim receive mismatch | 0.438 | 0.085 to 0.699 | 25 |
| Absolute WM qWC difference | −0.229 | −0.588 to 0.213 | 25 |
| Absolute GM qWC difference | −0.141 | −0.535 to 0.307 | 25 |

# 6 Supplementary Table S6. Scale and normalization trace.

The trace uses the verified primary-analysis outputs. Receive-field medians and ratios are participant-level summaries on the scale-trace support. The values document the executed scale convention; they are not a causal partition of every residual effect.

| **Quantity** | **Value** | **Unit** |
| --- | --- | --- |
| Mean median deployed *G*_Rx,_*_E_* | 1.076178 | dimensionless |
| Mean median deployed *G*_Rx,_*_T_* | 1.012541 | dimensionless |
| Mean ratio of medians *G*_Rx,_*_T_*/*G*_Rx,_*_E_* | 0.941135 | dimensionless |
| Cohort-median ratio *G*_Rx,_*_T_*/*G*_Rx,_*_E_* | 0.938790 | dimensionless |
| Mean inverse-divisor shift | +6.290 | % |
| Median inverse-divisor shift | +6.520 | % |
| Mean PDC change (participant-level median PDC) | +6.521 | % |
| Mean pre-CSF numerator change | +6.202 | % |
| Mean ventricular-CSF scale change | +6.349 | % |
| Residual median-normalized spatial mismatch | 2.259 | % |
| Mean final whole-brain qWC difference | −0.117 | p.u. |

# 7 Supplementary Figure S1. Brain-relative template-coordinate construction.

Source receive fields are restricted to the SPM-derived brain support, cropped to each participant's array-index brain bounding box, median-normalized, and resampled to a 48 × 48 × 32 brain-relative grid. The exact other-24 arithmetic mean is fitted with a fixed degree-3 polynomial and evaluated in the held-out participant's brain-relative frame before native deployment and qWC processing. Relative array-axis order, brain-relative location, and held-out native deployment geometry are preserved. Absolute scanner translation, physical scale, voxel spacing, DICOM positioning/orientation, and participant-specific coil geometry are not explicitly represented in the fitting coordinates.

# 8 Supplementary Figure S2. Descriptive normalized-grid Arm-T minus Arm-E qWC differences.

Three representative axial slices of the 32-slice normalized grid are shown (inferior, middle, and superior; slice indices 8, 15, and 22). Panels display (i) the cohort median signed Arm-T minus Arm-E qWC difference, (ii) the cohort median absolute difference, and (iii) participant contribution. Difference maps are shown only at locations contributed by at least 80% of participants; locations below this threshold are shown in gray. In panel (i), values near zero map to the white center of the diverging color scale and should not be interpreted as missing data. Panel (iii) shows the full 0–100% participant-contribution range. The maps are descriptive; they are not significance maps and do not treat voxels as independent observations.
